# Complex Glycerol Kinase Deficiency and Adrenocortical Insufficiency in Two Neonates

**DOI:** 10.4274/jcrpe.2539

**Published:** 2016-12-01

**Authors:** Sabriye Korkut, Osman Baştuğ, Margarita Raygada, Nihal Hatipoğlu, Selim Kurtoğlu, Mustafa Kendirci, Charalampos Lyssikatos, Constantine A. Stratakis

**Affiliations:** 1 Erciyes University Faculty of Medicine, Department of Pediatrics, Division of Neonatology, Kayseri, Turkey; 2 Eunice Kennedy Shriver National Institute of Child Health and Human Development, National Institutes of Health, Section on Endocrinology and Genetics, Program on Developmental Endocrinology and Genetics and Pediatric Endocrinology Inter-institute Training Program, Bethesda, Maryland, USA; 3 Erciyes University Faculty of Medicine, Department of Pediatrics, Division of Pediatric Endocrinology, Kayseri, Turkey; 4 Erciyes University Faculty of Medicine, Department of Pediatrics, Division of Pediatric Metabolism, Kayseri, Turkey

**Keywords:** Deletions, X-chromosome, glycerol kinase, adrenal insufficiency

## Abstract

Contiguous gene deletions of chromosome Xp21 can lead to glycerol kinase deficiency and severe adrenocortical insufficiency (AI) in a male newborn among other problems. We describe our experience with two such patients who presented with dysmorphic facies, AI, and pseudo-hypertriglyceridemia. Both infants had normal serum 17-hidroxyprogesterone levels, and adrenal glands could not be observed with ultrasonography. Creatine kinase and triglyceride levels were measured to elucidate the etiology of adrenal hypoplasia and were above normal limits in both cases. Both patients required steroid and salt supplementation. They were both found to have Xp21.2 deletions (DMD, NR0B1, GK, IL1RAPL1). We conclude that AI in the context of other genetic abnormalities should prompt chromosomal investigations in the absence of another unifying explanation.

**WHAT IS ALREADY KNOWN ON THIS TOPIC?**

Complex glycerol kinase deficiency (CGKD) typically develops from partial deletion of the Xp21 chromosomal locus involving the genes responsible for glycerol kinase deficiency, adrenal hypoplasia congenita, Duchenne muscular dystrophy, and others causing various developmental defects.

**WHAT THIS STUDY ADDS?**

CGKD is a rare disorder. We reported our experience in two neonates with CGKD.

## INTRODUCTION

Complex glycerol kinase deficiency (CGKD) is a contiguous gene deletion syndrome which is inherited as an X-linked trait. CGKD typically develops from partial deletion of the Xp21 chromosomal locus involving the genes responsible for glycerol kinase deficiency (GKD), adrenal hypoplasia congenita (AHC), Duchenne muscular dystrophy (DMD), and others causing various developmental defects. Symptoms are related to the extent of the deletion and may present early in life. The diagnosis is based on clinical and laboratory findings. With genetic analyses, it is possible to confirm the diagnosis by demonstrating gene deletion at Xp21 locus and the female carrier can be identified (1,2,3). We describe our experience with two such patients.

## CASE REPORTS

### Case 1

**Figure 1:**
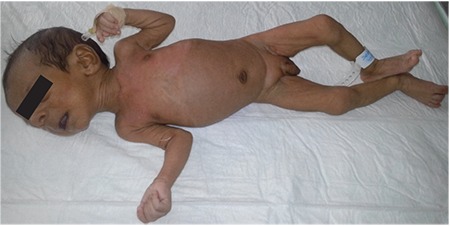
Hyperpigmented, dehydrated, and cachectic appearance (case 1)

**Figure 2:**
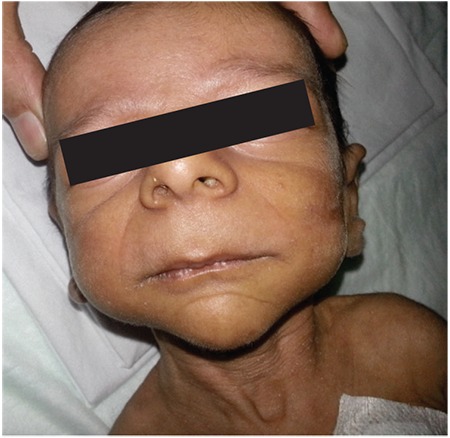
Dysmorphic facial features characterized by midfacial hourglass appearance, hypertelorism, long philtrum, rounded palpebral fissures (case 1)

**Figure 3:**
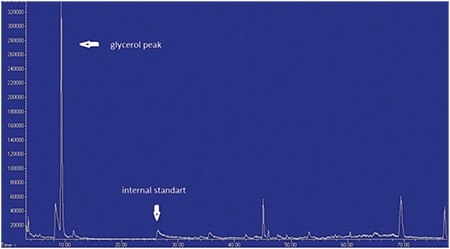
Glycerol peak against internal standards observed in urinary organic acid analysis (case 1)

**Figure 4:**
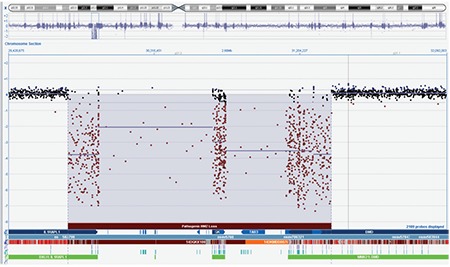
The deletion involving all coding seqeunces of the GK gene, including part of the DMD gene, the entire NR0B1 gene, and part of the IL1RAPL1 gene (case 1)

A 36-day-old male infant was brought to the hospital for difficulty to feed, vomiting, and weight loss. He was delivered at term and with no complications via normal vaginal delivery to a 36-year-old mother. There was no parental consanguinity; however, the second child of this couple had similar findings to our case and had died at 7 months of age because of muscle disease. The birth weight of our patient was 3200 g, but at the time of presentation, his weight was only 2700 g (<3^rd^ percentile). His length was 54 cm (50^th^ percentile) and head circumference was 38 cm (25^th^-50^th^ percentile). The infant was hypotonic, lethargic, and appeared to be malnourished and dehydrated. His skin was hyperpigmented, with pigmentation being more pronounced in the areola of the breasts and in the scrotum (Figure 1). He had dysmorphic facial features (Figure 2). Initial laboratory tests revealed the following serum levels: glucose: 57 mg/dL, sodium: 128 mEq/L, potassium: 8.6 mEq/L, serum cortisol: 12.6 µg/dL, adrenocorticotropic hormone (ACTH): >2000 pg/mL, 17-hydroxyprogesterone (17-OHP): 0.79 ng/mL. Based on these findings, the patient was considered to have partially compensated adrenocortical insufficiency. Fluid and electrolyte therapy along with hydrocortisone and fludrocortisone replacement at proper doses were initiated. The patient, who improved with treatment, was investigated for etiology. The adrenal gland could not be visualized by ultrasonography. Serum creatine phosphokinase (CPK) and triglycerides were investigated to evaluate complex glycerol kinase (GK) deficiency and were measured as 5758 U/L (normal range: 68-580) and 1193 mg/dL(normal range: 35-110), respectively. With urinary organic acid analysis using gas chromatography-mass spectrometry, the patient’s urinary glycerol excretion was 4847.6 mmol/mmol creatine (normal range: 0-40) (Figure 3). Routine peripheral lymphocyte chromosome analysis result was 46,XY. Comparative genomic hybridization (CGH) showed a deletion involving all coding seqeunces of the GK gene. The deletion included part of the DMD gene, the entire NR0B1 gene, and part of the IL1RAPL1 gene (Figure 4). On the 51^th^ day of hospitalization, the patient was discharged with oral hydrocortisone, fludrocortisone, and salt supplementation.

### Case 2

**Figure 5:**
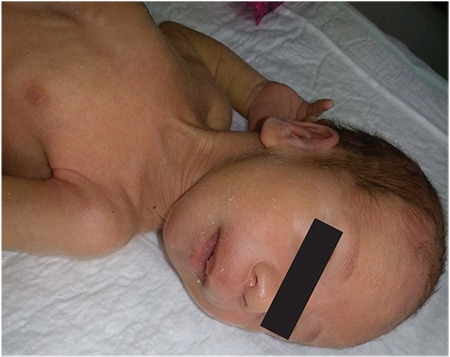
Dysmorfic facial features characterized by prominent forehead, rounded palpebral fissures, expanded and flattened ear lobes and long philtrum (case 2)

**Figure 6:**
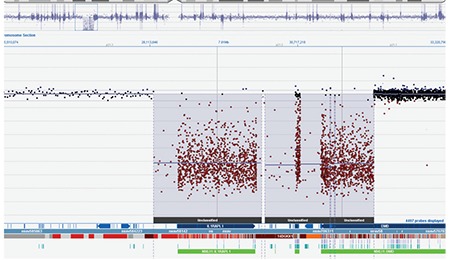
The deletions involving DMD, NR0B1, GK, and IL1RAPL1 genes (case 2)

A male infant delivered at term at another facility to a 33-year-old primigravida was brought to medical attention on the 18^th^ postnatal day for reduced breastfeeding, vomiting, and weight loss. There was no history of parental consanguinity. There were no similar cases in the pedigree. His birth weight was 3100 g, but at presentation, his weight was 2400 g (<3rd percentile). His length was 52 cm (25^th^-50^th^ percentile) and head circumference 37 cm (50^th^ percentile). The infant had dysmorphic facial features and was dehydrated (Figure 5). Laboratory values included serum glucose: 52 mg/dL, sodium: 124 mmol/L, potassium: 7.4 mmol/L, ACTH: 628 pg/mL, cortisol: 20.6 µg/dL, 17-OHP: 6.04 ng/mL. The adrenal glands could not be visualized by ultrasonography. Fluid and electrolyte therapy along with hydrocortisone and fludrocortisone replacement were initiated. Serum triglyceride level was 761 mg/dL, CPK was 28.134 U/L, and CK-MB was 592 U/L (normal range: 0-25). Routine karyotype was consistent with normal 46,XY constitution; however, CGH showed a 3.88 Mb deletion encompassing part of the DMD gene (exon 45 extending through 3’ end) and three additional disease-associated genes (NR0B1, GK, and IL1RAPL1) (Figure 6).

The patient was discharged with oral hydrocortisone, fludrocortisone, and salt supplementation when 42 days old.

Informed consent was obtained from the parents of the two children studied for further investigation. DNA was extracted by standard methodology and CGH.

## DISCUSSION

oth patients presented here were diagnosed with at least partially compensated primary adrenal insufficiency due to the lack of adequate elevation in cortisol levels, despite increasing ACTH levels, and presence of dehydration, hyponatremia, hyperpotassemia, and hyperpigmentation.

Congenital adrenal hyperplasia (CAH) is the most common cause of primary adrenal insufficiency. However, 17-OHP values below <10 ng/mL during the neonatal period rule out CAH (4). CAH is also associated with large adrenal glands at ultrasonography (5). The findings in our patients were supportive of AHC, as seen in patients with mutations or deletions of the DAX-1 (NROB1) gene at the X chromosome (6,7), defects of steroidogenic factor 1 gene at the 9q33 chromosome (8), and IMAGe syndrome (9). In X-linked AHC, deletions of the DAX-1 gene may occur along with deletions of adjacent genes at the Xp21 locus. In some cases, this may be accompanied by the deletion of the gene encoding dystrophin, leading to DMD. Other cases involve deletion of the GK leading to GKD. Thus, AHC manifestations vary depending on the site and extent of the deletion (2). When GKD is accompanied by DMD or AHC or both, this is called CGKD (1).

Creatine kinase and triglyceride levels were measured to elucidate the etiology of AHC and were above normal limits in both cases. Glycerol is measured as triglyceride in routine laboratory tests. Thus, elevated levels of triglycerides in these cases are not described as hypertriglyceridemia but, as a more precise term, as “pseudo-hypertriglyceridemia” (10). Although glycerol is not an acidic compound, glyceroluria can usually be detected with urinary organic acid measurements using gas chromatography-mass spectrometry (1). There was a glycerol peak in the urinary organic acid assay in case 1. With these findings, both of our cases were considered as CGKD with coexisting AHC, DMD, and GKD.

Deletions and mutations in the DMD, AHC (NROB1), and GK genes at locus Xp21 can be demonstrated by genetic analysis in CGKD. CGH showed a deletion involving all coding seqeunces of the GK gene, the deletion included part of the DMD gene, the entire NR0B1 gene, and part of the IL1RAPL1 gene in the first patient. CGH also showed a deletion encompassing part of the DMD and three additional disease-associated genes (NR0B1, GK, and IL1RAPL1) in case 2.

Patients with concurrent AHC, DMD, and GKD have characteristic facial features. These include prominent forehead and eyebrows, depressed nasal root and bridge, which together give an “hourglass” appearance to the midfacial region. Other facial characteristics are hypertelorism, rounded palpebral fissures, esotropia, wide and flattened ear lobes, and downturned corners of the mouth (11).

In conclusion, AHC and CGKD should be considered in male neonates with dysmorphic features presenting with adrenal crisis. Performing genetic analysis such as CGH is helpful in finalizing the diagnosis and predicting prognosis by determining the location and magnitude of deletions as well as in detection of female carriers.

## Ethics

Informed Consent: It was taken.

Peer-review: Externally peer-reviewed.

## References

[1] Dipple KM, Mccabe ERB, Blau N, Duran M, Blaskovics ME, Gibson KM (2003). Disorders of glycerol metabolism. Physician’s Guide to the Laboratory Diagnosis of Metabolic Diseases.

[2] Wikiera B, Jakubiak A, Zimowski J, Noczynska A, Smigiel R (2012). Complex glycerol kinase deficiency-X-linked contiguous gene syndrome involving congenital adrenal hypoplasia, glycerol kinase deficiency, muscular Duchenne dystrophy and intellectual disability (IL1RAPL gene deletion). Pediatr Endocrinol Diabetes Metab.

[3] Ramanjam V, Delport S, Wilmshurst JM (2010). The diagnostic difficulties of complex glycerol kinase deficiency. J Child Neurol.

[4] Working Group on Neonatal Screening of the European Society for Paediatric E (2001). Procedure for neonatal screening for congenital adrenal hyperplasia due to 21-hydroxylase deficiency. Horm Res.

[5] Al-Alwan I, Navarro O, Daneman D, Daneman A (1999). Clinical utility of adrenal ultrasonography in the diagnosis of congenital adrenal hyperplasia. J Pediatr.

[6] Habiby RL, Boepple P, Nachtigall L, Sluss PM, Crowley WF, Jameson JL (1996). Adrenal hypoplasia congenita with hypogonadotropic hypogonadism: evidence that DAX-1 mutations lead to combined hypothalmic and pituitary defects in gonadotropin production. J Clin Invest.

[7] McCabe ER (2007). DAX1: Increasing complexity in the roles of this novel nuclear receptor. Mol Cell Endocrinol.

[8] Achermann JC, Ito M, Ito M, Hindmarsh PC, Jameson JL (1999). A mutation in the gene encoding steroidogenic factor-1 causes XY sex reversal and adrenal failure in humans. Nat Genet.

[9] Bergada I, Del Rey G, Lapunzina P, Bergada C, Fellous M, Copelli S (2005). Familial occurrence of the IMAGe association: additional clinical variants and a proposed mode of inheritance. J Clin Endocrinol Metab.

[10] Goussault Y, Turpin E, Neel D, Dreux C, Chanu B, Bakir R, Rouffy J (1982). ‘Pseudohypertriglyceridemia’ caused by hyperglycerolemia due to congenital enzyme deficiency. Clin Chim Acta.

[11] Scheuerle A, Greenberg F, McCabe ER (1995). Dysmorphic features in patients with complex glycerol kinase deficiency. J Pediatr.

